# Central serous chorioretinopathy: updates in the pathogenesis, diagnosis and therapeutic strategies

**DOI:** 10.1186/s40662-023-00349-y

**Published:** 2023-07-11

**Authors:** Xinyuan Zhang, Connie Zhi Fong Lim, Jay Chhablani, Yew Meng Wong

**Affiliations:** 1 Beijing Tongren Eye Center, Beijing Institute of Ophthalmology, Tongren Hospital, Capital Medical University, Beijing University of Medical Science, Beijing, 100730 People’s Republic of China; 2 UPMC Eye Center, University of Pittsburgh, Pittsburgh, USA; 3 Southern Specialist Eye Center SDN, BHD, Malacca, Malaysia

**Keywords:** Central serous chorioretinopathy, Epidemiology, Pathogenesis, Diagnosis, Multimodality imaging, Treatment

## Abstract

Central serous chorioretinopathy (CSCR), first described by Albrecht von Graefe in 1866, is characterized by focal serous detachment of the neural retina and/or retinal pigment epithelium (RPE) in the posterior pole. CSCR is the first ever described pachychoroid disease. Most recently, hypothetical venous overload choroidopathy is also proposed due to its distinguished morphological and pathological characteristics, including choroidal thickening, choriocapillaris hyperpermeability, remodelling, and intervortex venous anastomoses. Identification of genetic variants is necessary to comprehend the pathophysiology of CSCR. The novel multimodality imaging platforms, including the ultra-widefield imaging system, flavoprotein fluorescence, fluorescence lifetime imaging ophthalmoscopy, and multispectral imaging system, have been used for diagnosing and managing CSCR. Half-dose photodynamic therapy (PDT) remains the mainstay of clinical practice, with about 95% of patients with chronic CSCR improving to visual acuity (VA) of 20/30 or better. The use of oral eplerenone for routine clinical care remains controversial, and long-term randomized clinical trials are warranted to investigate its efficacy in acute and chronic CSCR. While CSCR has generally been recognized as a self-limiting disease with good prognosis, the underlying pathogenesis is still not fully understood, and treatments are often not fully effective. With new evidence emerging about pachydrusen being a disease precursor in both CSCR and polypoidal choroidal vasculopathy (PCV), it would be interesting to investigate whether CSCR can be a precursor to PCV. In this review, we highlighted the currently available evidence on the pathogenesis, diagnosis, multimodality imaging features, and management strategies, including recent findings related to CSCR.

## Background

Central serous chorioretinopathy (CSCR) is characterized by idiopathic focal serous detachment of the neural retina and/or retinal pigment epithelium (RPE) in the posterior pole. Acute CSCR (aCSCR) is usually self-limiting, but permanent visual impairment can be found in some chronic CSCR (cCSCR) patients due to photoreceptor damage or atrophy of the RPE. CSCR was initially introduced by Albrecht von Graefe in 1866 with the term “central recurrent retinitis.” Various labels have been used to describe this condition, including idiopathic central serous choroidopathy, central serous pigment epitheliopathy, central serous retinopathy, and the currently favoured term, central serous chorioretinopathy which was coined by Donald Gass in 1967 [1–3]. Since then, both nomenclature and physiopathology have evolved and numerous discoveries have been made in the fundamental understanding of its physiopathology.

In the recent 15 years, with the development of advanced imaging techniques such as optical coherence tomography (OCT), a novel concept of pachychoroid spectrum disorder (PCD), has been described. CSCR is the first entity being described and is one of the most common phenotypes of PCD. It is characterized by chronic persistent thickening of the choroidal layer and dysfunction of the choriocapillaris, with or without RPE abnormalities overlying the pachyvessels as detected by optical coherence tomography angiography (OCTA) [4]. Most recently, venous overload choroidopathy was also proposed by Spaide, who noticed that CSCR shares the same pathological characteristics with carotid-cavernous sinus fistulas (CCSF) and peripapillary pachychoroid syndrome, including delayed choroidal filling, dilated veins, intervortex venous anastomoses and choroidal vascular hyperpermeability [5].

The treatment of CSCR aims to restore anatomical and functional photoreceptor-RPE interaction, improve choroidal perfusion, and achieve complete resolution of the neurosensory. Compared to laser photocoagulation (LP), photodynamic therapy (PDT), which induces choriocapillaris vascular remodelling and decreases choroidal hyperpermeability, has been proven safer and more efficacious for patients with cCSCR [6]. Oral therapy such as mineralocorticoid antagonists, antibiotics, and anti-tubercular drugs have been introduced in the treatment of CSCR for patients who may need to use non-invasive treatment or who are not candidates for invasive modality. Subthreshold micropulse laser (SML) photocoagulation has also been proven to be effective in the lack of photosensitizer of PDT, although it has limited effects on choroidal vascular hyperpermeability.

In this review, we aim to highlight the currently available evidence on the pathogenesis, diagnosis, multimodality imaging features, and management strategies, including recent findings related to CSCR.

## Literature search

In this review, PubMed, Medline, Springerlink, the Cochrane Library, Google Scholar, and EMbase Medline database from the year 1990 to November 28, 2022, have been searched to retrieve English articles with the following key terms: "central serous chorioretinopathy", "central serous retinopathy", "chorioretinopathy", "central serous retinopathy" and "CSCR". The search is limited to the terms "epidemiology" or "risk factors" or "pathogenesis"; or "management" or "treatment" or "trial" or "randomized" to focus on the findings of randomized clinical trials, cohort studies, meta-analyses, reviews, and technological advancements in the past decade.

## Epidemiology

CSCR is one of the most common retinal diseases that could lead to visual impairment, being ranked fourth in incidence among the non-surgical retinal diseases with subretinal fluid (SRF), after age-related macular degeneration (AMD), diabetic retinopathy (DR) and retinal vein occlusion (RVO). To date, the epidemiology of CSCR lacks a systematic survey. It occurs predominantly in middle-aged adults between 30 to 50 years old, and more often, it affects men with a heavy workload [7, 8]. The annual incidence in men is approximately 10 per 100,000. Various studies have reported a higher prevalence of CSCR in men than in women in clinic-based populations, with men accounting for 72% to 87.5% of CSCR patients [9, 10]. However, when stratified by age, older women presented a similar prevalence rate as men, indicating that CSCR in women could be severe and evolve toward chronic forms. In addition, there appeared to be racial predisposition in the variation of CSCR incidence, in which CSCR is suspected to be more prevalent in Asians when compared to Caucasians and African Americans [11].

## Risk factors

### Glucocorticoids

Glucocorticoids are well-known and related to the pathogenesis of CSCR. It was reported that in South Korea, from the year 2011 to 2015, the prevalence was 9.4 in men and 3.0 in women per 10,000 corticosteroid users, and the annual prevalence gradually increased from 5.5 in 2011 to 7.3 in 2015 (per 10,000 persons) in population-based studies [12]. High levels of glucocorticoids, mineralocorticoids, and testosterone have been found in patients with CSCR. Further research has also confirmed the association of steroids in the pathogenesis of CSCR in patients with Cushing’s syndrome [13].

It has been demonstrated that not just endogenous steroids but all forms of exogenous corticosteroid administration, including oral, nasal, topical, intravenous, and intravitreal, seem to be significantly associated with an increased risk of precipitation, aggravation and even cause a relapse of CSCR [14]. From a multicenter survey, cases with corticosteroid treatment were found to be of an older age group, had more bilateral involvement, and multiple pigment epithelial detachment in the affected eyes, more fluorescein leakage sites, greater choroidal thickness, and a higher recurrence rate [15]. Topical and inhaled corticosteroids are also associated with CSCR in several case studies [16, 17]. Additionally, high levels of endogenous corticosteroids during pregnancy can increase the risk of CSCR. Ocular signs usually appear during the third trimester and resolve spontaneously after delivery. Many studies have suggested that glucocorticoids have an essential impact on the RPE, Bruch’s membrane, and the choriocapillaris, possibly altering their permeability directly or secondarily due to vascular autoregulation or inhibition of collagen synthesis, aggravating SRF accumulation, and thus lead to the development of CSCR [18].

Overactivation of the retinal or choroidal mineralocorticoid receptor pathway has been proposed to contribute to CSCR. Recent pre-clinical and clinical evidence also indicates that oral intake of mineralocorticoid receptor agonists can effectively treat CSCR [19–21].

### Stress

Psychological and psychosocial stress should be given attention during the diagnosis and treatment of CSCR. Several clinical studies pointed to an association between CSCR and personality traits, with type A personality being most at risk [1]. This describes a person characterized by a sense of urgency, competitiveness, and aggressiveness with a hostile temperament [1]. The mechanistic link between CSCR and personality factors is proposed to be mediated through the activation of the neuroendocrine system with the release of catecholamines and corticosteroids, leading to changes in choroidal vascular permeability [22]. Spaide proposed an alternative explanation to the underlying mechanism of psychological stress on CSCR: an increase in cortisol which acts as a sympathetic antagonist leads to the dilation of the choroidal veins under pressure. On the other hand, the consequent increase of the parasympathetic tonus increases the flux to the choroid and leads to the loss of the capability to adapt to raises in systemic arterial tension related to stress that is dependent on sympathetic regulation [23]. However, a meta-analysis concluded that CSCR patients have significant overactivation and decreased parasympathetic activity compared to normal healthy subjects [14]. Therefore, validation of this discrepancy is warranted through the study of large-scale cohorts and preclinical studies.

Severe psychological stress and anti-psychotropic medication were independent risk factors for the onset of CSCR [24]. Depression is associated with an increased risk of recurrent CSCR [9]. Besides, poor sleep quality and anxiety also contribute to CSCR. Patients with CSCR are more likely to have poorer sleep quality (58.2% versus 23.9%) and greater chances of developing negative emotions such as stress (23.9% versus 3.0%), depression (25.4% versus 10.4%) and anxiety (28.4% versus 14.9%) when compared with normal controls. This study assessed certain specific components of sleep quality (such as sleep latency, sleep duration, and sleep efficiency) in patients with CSCR using the Pittsburgh sleep quality index (PSQI) [25]. Obstructive sleep apnoea is also closely correlated with the increased risk of CSCR [26]. Thus, attention should be paid to patients’ sleep issues and emotions to achieve better treatment outcomes.

### Catecholamines

It is believed that there is a correlation between the use of sympathomimetic agents [such as pseudoephedrine, oxymetazoline, 3-methoxy-4,5-methylenedioxyamphetamine (MMDA)] and CSCR [27]. A meta-analysis concluded that CSCR patients have a significant overactivation and decreased parasympathetic activity compared to normal healthy subjects [14, 28]. The plasma concentration of catecholamine (epinephrine and norepinephrine) rises significantly in the active stage of CSCR. It normalizes in the convalescent stage, indicating that plasma epinephrine was strongly associated with SRF, playing a significant role in the pathogenesis of CSCR [29]. The above explanation may contradict Spaide’s statements so further studies are warranted.

### *Helicobacter pylori* infection

Some researchers have postulated that *H. pylori* infection, which is highly correlated with visual acuity (VA) and the clinical severity of the disease, is a risk factor for CSCR. *H. pylori* infection is deemed to cause the retinal tissue to be more sensitive to inflammatory reactions triggered by oxidative stress and to reduce cellular antioxidant capacity, but the exact mechanism remains unknown [30].

### Miscellaneous

Other risk factors, including untreated hypertension, use of alcohol, pregnancy, kidney disease (type II membranoproliferative glomerulonephritis), and genetic factors such as age-related maculopathy susceptibility 2 (*ARMS*2) and complement factor H (*CFH*) have also been proposed in various studies and showed significant association with the occurrence of CSCR [14, 19, 31]. However, conclusions cannot be drawn due to insufficient evidence. Further studies are needed to understand the relationship between these risk factors and CSCR.

## Pathogenesis

The exact pathogenesis of CSCR is yet to be established. Many investigators hypothesized that the pathological event of CSCR is in either or both the choroidal vasculature and RPE.

In recent years, clinical studies have found that the primary site of pathology is within the choroid [9]. Impaired autoregulation of choroidal circulation is thought to be associated with glucocorticoids- or catecholamine-induced CSCR [32]. Dysautoregulation of choroidal circulation may lead to choroidal vascular hyperpermeability and abnormal accumulation of fluid beneath the RPE, causing an increase in choroidal hydrostatic pressure [9, 33]. High hydrostatic pressure pushes the fluid out of the choroidal vascular system and leads to the disintegration of the RPE, which in turn results in serous retinal detachment (SRD) [34]. Lesions of the RPE can be visualized by observing the areas of fluorescein leakage using FA and are often referred to as "microrips" or "blowouts" [35, 36].

Further evidence supporting the role of choroidal hyperpermeability in CSCR is based on the use of enhanced depth imaging OCT (EDI-OCT) or swept-source OCT (SS-OCT). OCT has revealed the thickening of the subfoveal choroid layer in CSCR patients. Studies have also concluded that the subfoveal choroidal layer was thicker in patients with CSCR compared with the normal eyes [37, 38]. A study that evaluated choroidal thickness in patients with unilateral CSCR found that choroidal thickness was increased in both the affected eyes and the unaffected fellow eyes in comparison with the normal control in both groups, the choroidal thickness was significantly greater in the eyes with active CSCR than in the fellow affected eyes [37].

The pachychoroid disease spectrum is a relatively new concept characterized by similar clinical and histopathological features: chronic persistent thickening and dysfunction of the choroidal vessels. Seven entities, including pachychoroid pigment epitheliopathy, CSCR, pachychoroid neovasculopathy, polypoidal choroidal vasculopathy (PCV)/aneurysmal type 1 neovascularization, focal choroidal excavation, and peripapillary pachychoroid syndrome are distinguished as PCD. CSCR was the first recognized PCD in the pachychoroid spectrum. On OCT, there is a focal or diffuse increase in choroidal thickness, accounted for by dilated choroidal vessels in the outermost choroid layer (Haller’s layer), together with the thinning of the Sattler’s layer and attenuation of choriocapillaris [39]. Several studies have drawn a correlation between the occurrence of CSCR and pachydrusen—a new entity characterized by isolated or scattered, well-defined boundary drusen throughout the posterior pole accompanied by thickened choroid. In a retrospective study, pachydrusen was found to be the indicator of choroidal circulation impairment and also a feature of chronic persistent or resolved CSCR [40].

The genetic corroboration of the pachychoroid concept also offers new perspectives to interpret the imaging findings. cCSCR, PCV and AMD may share similar genetic and pathophysiologic characteristics, as proven by some studies which have shown that cCSCR is associated with genetic variants in *ARMS*2 and *CFH*. There are significant disparities in allele frequencies among the phenotypic subgroups of the tested single nucleotide polymorphisms (SNPs) which are also associated with AMD. They found that certain SNPs of the *CFH* showed opposite effects between cCSCR and AMD, with the SNPs that are protective against AMD conferring risk to patients with cCSCR [41, 42]. *ARMS2 A69S* and *CFH I62V* genotypes are believed to be used as prognostic markers in predicting the prognosis of CSCR, *CFH,* and *ARMS2*/*HTRA1* are also correlated with PCV and retinal angiomatous proliferation in both Asians and Caucasians [43]. These genetic findings may aid in determining the need for early treatment and possibly prevent the development of subsequent choroidal neovascularization (CNV). Further prospective genetic studies are needed to clarify the role of genetic elements in CSCR [44].

Most recently, the term ‘venous overload choroidopathy’ was coined to describe a group of diseases characterized by delayed choroidal filling, dilated veins, intervortex venous anastomosis, and choroidal vascular hyperpermeability as elucidated by SS-OCT and wide-field indocyanine green angiography (ICGA) [5]. As CSCR shares similar clinical features with carotid cavernous sinus fistulas and peripapillary pachychoroid syndrome, it is a pathophysiological model for venous overload choroidopathy. This new concept has provided insights into the diagnosis and treatment for this group of choroidal vessel dysfunction diseases.

## Clinical manifestations

CSCR is commonly unilateral; however, bilateral involvement occurs in 5%–18% of the cases and is mainly seen in elderly patients. Frequent symptoms include recurrent blurred vision with a relative central scotoma, often associated with micropsia, variable metamorphopsia, dyschromatopsia, hypermetropization, and reduced contrast sensitivity [3, 19]. CSCR commonly manifests unilaterally and can be found in OCT abnormalities including pachychoid or pachychoroid pigment epitheliopathy [4].

CSCR can be classified into two distinct clinical presentations: acute and chronic [9, 45]. aCSCR is clinically apparent in middle-aged men, and most cases are usually self-limiting. It often resolves spontaneously within 2–3 months. The prognosis for vision is highly dependent on baseline best corrected VA (BCVA). Several case studies have found that after recovery, patients with an initial VA of 6/6 would have similar VA as the premorbid values, while patients with an initial VA of less than 6/9 would decline to two to three Snellen lines in the next few years [46, 47]. Several factors may contribute to incomplete visual recovery: repeated relapses, unresolved neurosensory retinal detachment, retinal pigment epithelial detachment (PED), and severe cases of CSCR. aCSCR mainly occurs as a single episode over a lifetime. However, it can be recurrent and progress to cCSCR if the SRD lasts longer than 3–6 months [6]. Recurrent CSCR is defined as a new episode of aCSCR within 12 months of the previously resolved episode, with the characteristics of a localized, circumscribed area of retinal detachment, particularly within the macular region. The recurrence rate is expected to occur in 19%–51% of all cases [48, 49]. cCSCR, previously termed “diffuse retinal epitheliopathy” [50], is defined by the presence of persistent fluid accumulation as seen on OCT, with widespread RPE changes and extensive choroidal vasculopathy as revealed by fundus fluorescence angiography (FFA) and ICGA [51]. The persistent presence of SRF in cCSCR is believed to be a consequence of recurrences of CSCR and prolonged detachment of the neurosensory retina could lead to progressive deterioration of photoreceptors and RPE atrophy. Therefore, the prognosis for visual recovery is less favourable in both recurrent and cCSCR. Permanent loss of VA with decreased light sensitivity may develop because of the disease progression [19, 52]. Retrospective studies are critical to understanding the progression of CSCR from the acute to the chronic phase. However, although a duration of 3 to 6 months is often arbitrarily used in clinical practice, there is no clear time frame that defines CSCR as a chronic condition [3, 46]. The distinction is very important as cCSCR may not share the same pathophysiology as the aCSCR and may require a different approach in its management.

Recently, several CSCR categories have been proposed to better understand the disease prognosis and to define treatments. These have been termed recurrent CSCR, non-resolving CSCR and inactive CSCR [9]. Complications of CSCR include focal hyper- or hypo-pigmentation of the RPE, atrophy or hyperplasia of RPE, and CNV [53]. Follow-up studies have concluded that CNV secondary to cCSCR can occur either as the natural course of the disease or as a complication of focal treatments, with a prevalence rate of 2% to 15.6% [46, 54].

Recent multimodal imaging based CSCR classification provides a more objective manner for classifying CSCR. The presence or evidence of prior SRD in the posterior pole that is not associated with other diseases, at least one area of RPE alteration on FFA, OCT, or infrared imaging is suggested to be the major diagnostic criteria. Whereas ICGA/ FFA characteristics, the subfoveal choroidal thickness of more than 400 µm or greater (age and the refractive error should be taken into account) are recommended as the minor criteria [55]. A simple, complex, and atypical classification system based on a multimodal imaging platform has also been introduced. This classification is directly correlated with the intervention strategies and can be regarded as a significant step in describing the pathophysiological features of CSCR [55].

However, up till now, there is no universally accepted classification system for CSCR, the precise and optimized criteria may be based on phenotypical characteristics to improve management strategies.

## Multimodality imaging for CSCR

**Fig. 1:**
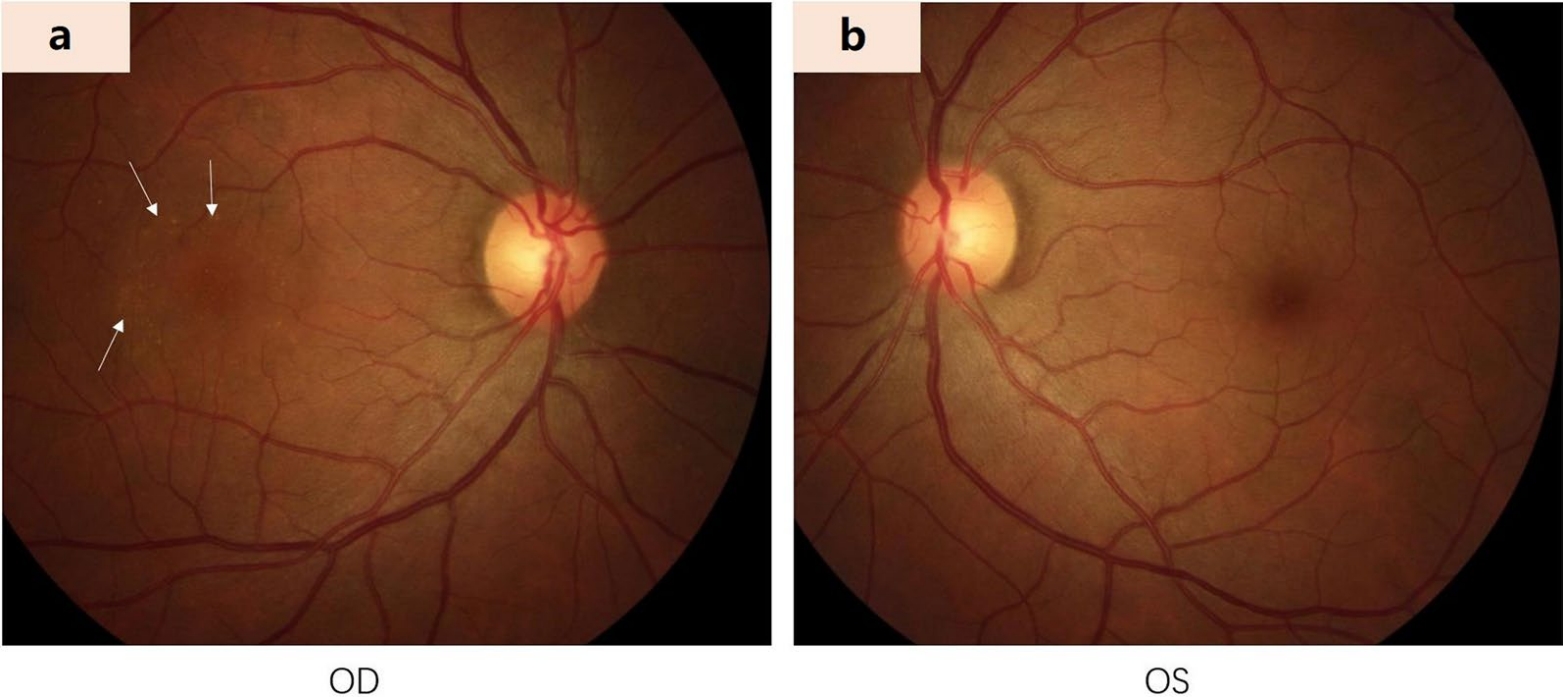
A 54-year-old female complained of blurred vision and metamorphopsia for two weeks. A colour fundus photo of the patient with central serous chorioretinopathy (CSCR) reveals several yellow pinpoint deposits (white arrows) on the temporal side of the foveal with subretinal shallow detachment in the macular (**a**, right eye). The fundus of the left eye is normal (**b**)

Various imaging modalities such as colour fundus photography (Fig. 1), OCT, ICGA, FFA, and fluorescein angiography (FA) are helpful in diagnosis and the management of CSCR [45, 56]. These imaging modalities have contributed to the understanding of the pathophysiology of CSCR. Furthermore, flavoprotein fluorescence (FPF) and multispectral imaging (MSI) systems have become essential auxiliary examination methods. A careful clinical examination and appropriate use of multimodal imaging are important to differentiate CSCR from other chorioretinal diseases [9]. Besides averting severe and permanent visual loss that may arise from misdiagnosis and inadequate management, this multimodal imaging can be utilized to detect several predictive biomarkers relevant to prognosis and treatment outcomes.

### Fundus fluorescein angiography

**Fig. 2:**
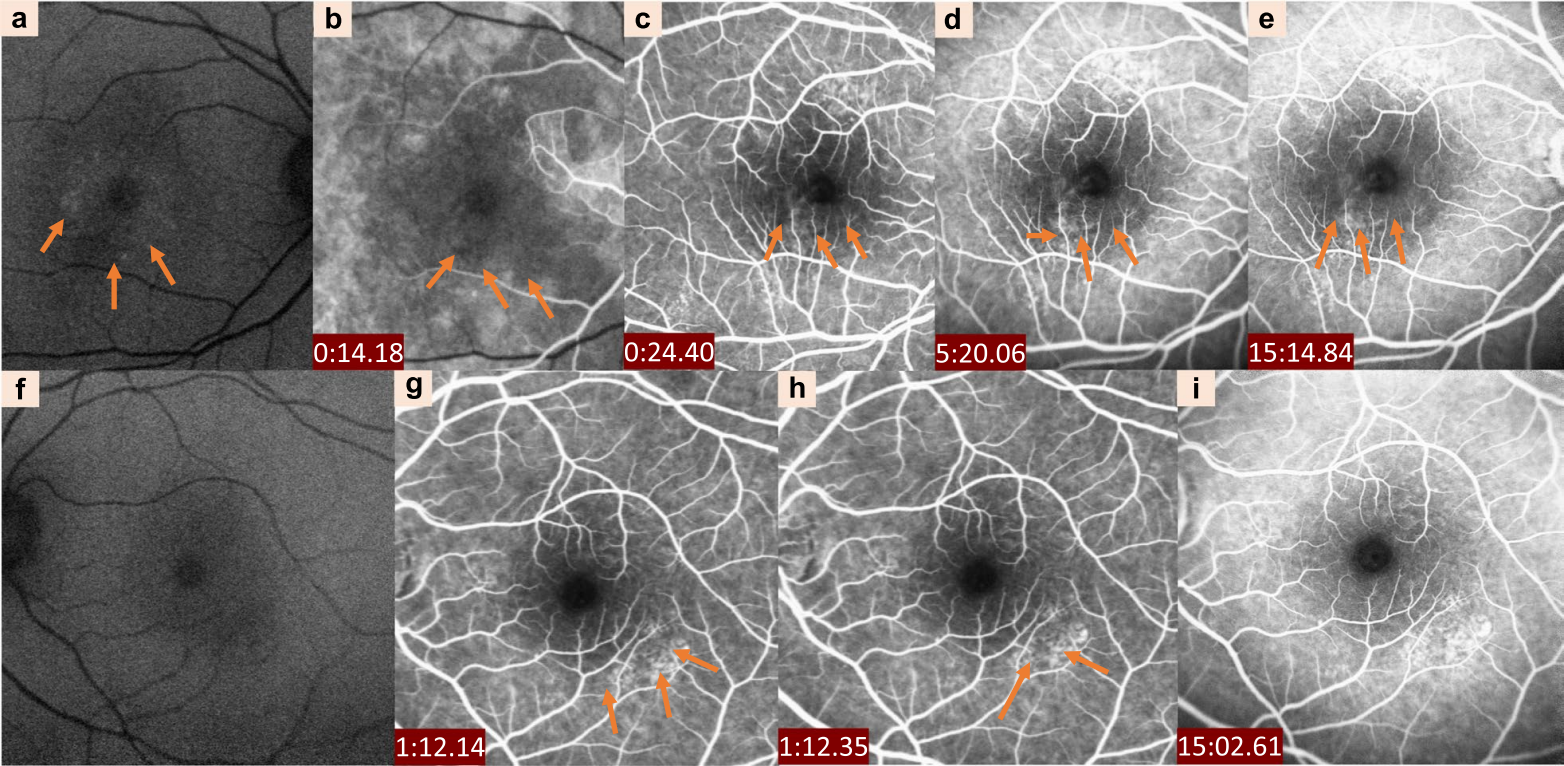
Clinical characteristics of central serous chorioretinopathy detected by fundus autofluorescence (FAF) and fundus fluorescence angiography (FFA). FAF shows pinpoint hypercyanescence in the macular area (**a**). FFA shows hyperfluorescent leakage points (orange arrows) at the lower part of the fovea in the early phase, with a progressive increase in intensity and size over time (**b**–**e**). Spot-like transparent fluorescence is visible at the posterior pole in the left eye (**f**–**i**). The hyperfluorescent leakage points (orange arrows) at the low part of the macular were also detected in the early phase in the left eye (**g**, **h**)

FFA is a long-established imaging modality for the evaluation of CSCR. In acute cases, there can be one or more leakage points at the level of RPE, underneath or adjacent to the area of SRF. More often than not, the leakage points are located within the macula [57]. Leakage begins as a pinpoint hypercyanescence dot in the early phase of FFA. It then evolves into two types of classical patterns in the mid and late phases, described as “ink-blot” (circular expansion) and “smoke-stack” (ascending expansion) (Fig. 2). The “ink-blot” pattern is more common (about 53% to 93% of cases), while the “smoke-stack” pattern is seen in only about 7% of the cases [58, 59]. Research has shown that patients with cCSCR have multifocal or diffuse leakage points in FFA, which are visible as patchy, granular or mottled hyper-fluorescence in the mid- and late-phases due to RPE defects [58]. Sometimes, affected eyes with CSCR occasionally manifest other atypical angiographic leakage patterns, including “blowout leak” and “leaking scar” [60, 61].

### Indocyanine green angiography

**Fig. 3:**
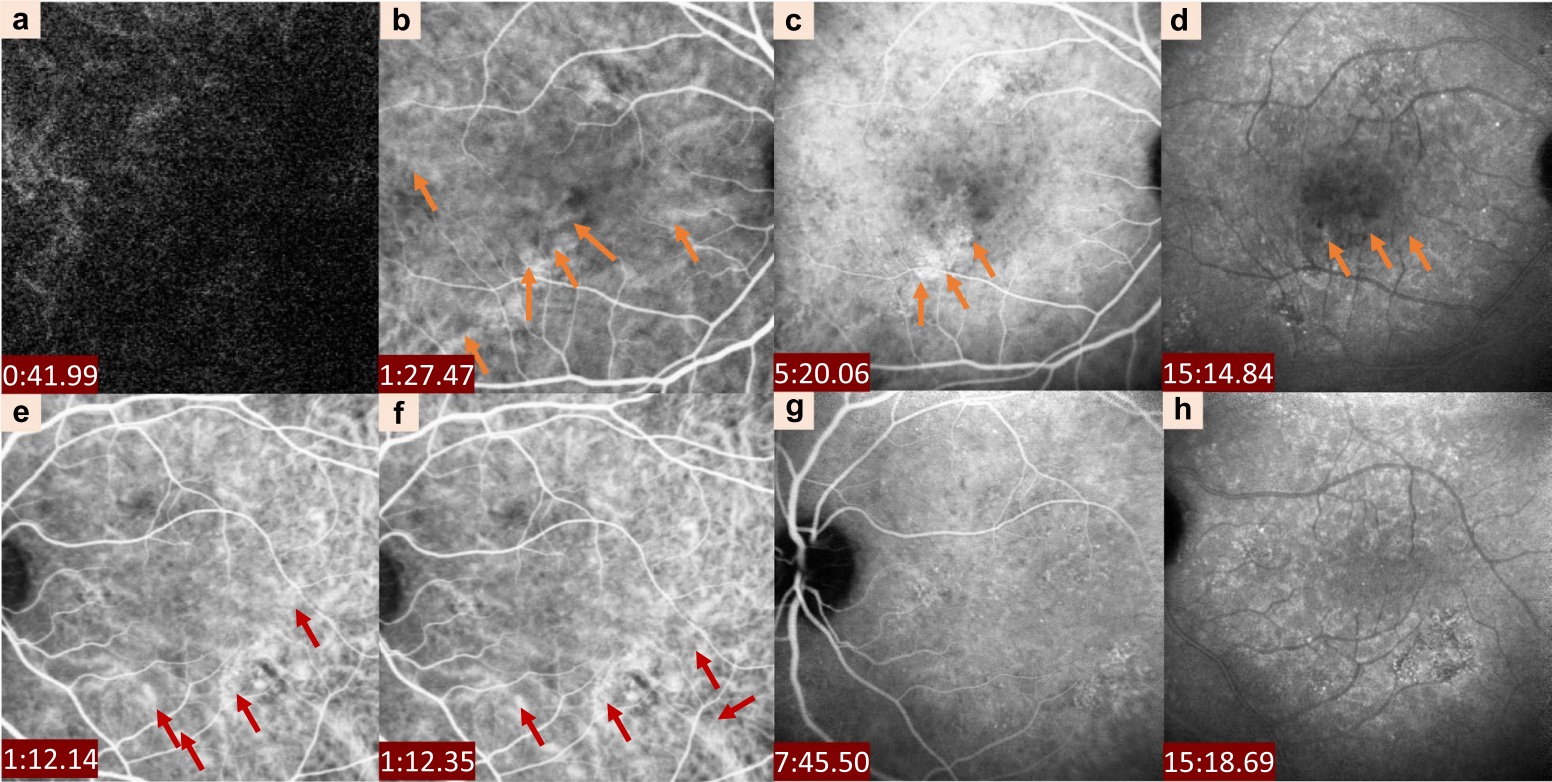
Clinical characteristics of central serous chorioretinopathy detected by indocyanine green angiography (ICGA). ICGA showing massive choroidal dye filling delay (**a**) and hyperfluorescent lesions in correspondence with the FFA findings (red arrows) in the left eye. Abnormal dilation of choroidal vessels and related choroidal hyperpermeability is found in the posterior pole (orange arrows) in the right eye (**a**–**d**). Dilated choroidal vessels are also visible at the posterior pole (red arrows) in the left eye (**e**–**h**)

ICGA remains the gold standard for imaging the choroidal vasculature, and differentiating CSCR from other retinal and choroidal diseases such as CNV or PCV [62]. It is particularly helpful in guiding PDT for CSCR. ICGA allows the visualization of abnormalities in the deeper layers of the choroid [63]. The main ICGA findings in most CSCR eyes are the presence of a delayed choroidal filling in the early phase [64], marked dilation of large choroidal vein, and multifocal choroidal hyper-fluorescence with blurred contours in the mid-phase which confirms status of choroidal vascular hyperpermeability [10]. This hypercyanescence fades or persists into the late phase [19] (see Fig. 3).

ICGA is a valuable diagnostic tool capable of pinpointing leakage areas in cCSCR. It can overcome several limitations of FA, such as complex analysis of leakage sites on FA images, and sometimes even false negative results when the leakage sites were undetectable on FA despite proven increased hyperpermeability in the choroidal vasculature [10, 64, 65]. ICGA is a valuable tool to evaluate the function of choroidal capillary vasculature precisely to improve our knowledge and management decisions. The Optos ultra-widefield (UWF) FA and ICGA (Dunfermline Scotland, UK) have increased our ability to capture the peripheral retina up to 200 degrees in a single image using light sources with emission wavelength of 488 nm for FA and 802 nm for ICGA, providing insights into the possibility of quantification on the high-contrast whole image of choroidal vessels [66, 67]. In normal eyes, the number of discrete vortex vein ampullae discernible by UFW ICGA is more significant than four, contrary to traditional thought [68]. The vessel density is significantly higher in eyes with CSCR throughout the entire area, as detected by UFW ICGA in the superior, posterior, and peripheral portions [66].

### Optical coherence tomography

OCT, which is based on low coherence interferometry and employs near-infrared light to produce two- and three-dimensional images, has been applied in the diagnosis and follow-up of CSCR [69, 70]. OCT allows for the detailed observation of the morphologic changes of retinal and choroid capillaries, identification of leakage site and disruption at the leakage site, which involve the single retinal photoreceptor layers and the alterations occurring in the Brunch membrane-RPE-choroidal capillaries complex and beyond [71].

**Fig. 4:**
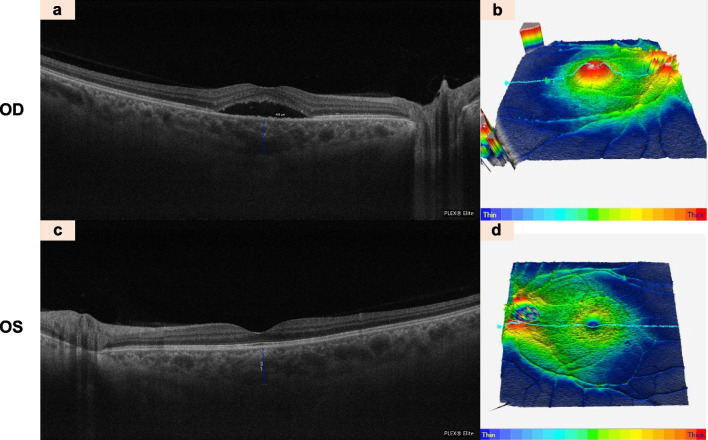
Clinical characteristics of central serous chorioretinopathy detected by Optical coherence tomography (OCT). OCT B-scan of the right eye showing visible subretinal fluid (low-density area) indicates the area of serous retinal detachment (**a**). OCT B-scan showing thickness map of the right eye (**b**). OCT B-scan of the left eye is normal (**c**, **d**). The choroidal thickness of both eyes was obviously increased, with 488 nm in the right eye and 450 nm in the left eye

**Fig. 5:**
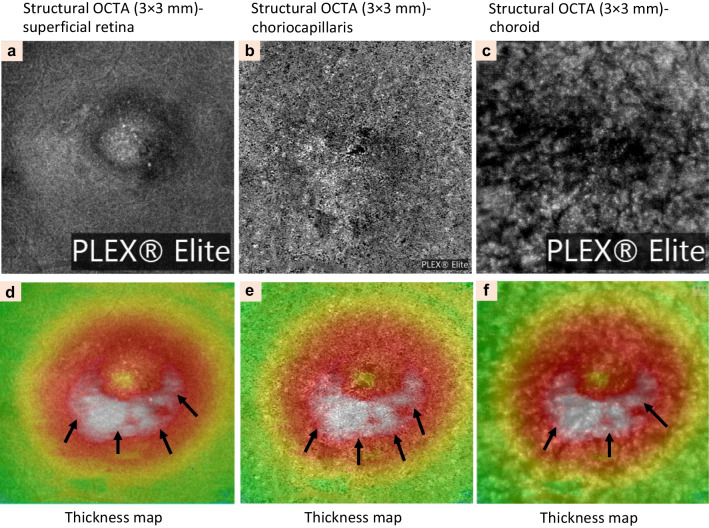
Clinical characteristics of central serous chorioretinopathy detected by structural *en-face* optical coherence tomography angiography (OCTA). Automated segmentation of the superficial retina (3 × 3 mm) showing dark spots under the fovea indicates pigmented epithelial detachment (PED) at the superficial, deep retinal capillary, and retina plexuses (**a**–**c**) of the left eye. Thickness map of automated segmentation images showing high-density area corresponding to the subretinal fluid (black arrows, **d**–**f**)

Furthermore, the emergence of EDI-OCT and SS-OCT provides improved visualization of the choroidal layer, enabling more accurate morphological analyses of the choroidal vasculature and choroidal thickness measurements [72]. It has been reported that increased choroidal thickness can be observed in both the affected and contralateral eyes of CSCR patients compared with normal healthy control subjects, and thus strengthen the original idea of a hyperpermeable choroid as a potential cause [19, 73, 74]. The thickening of choroidal layers results from the dilation of the outer choroidal vessels and attenuation of the inner choroidal layers (Fig. 4). Meanwhile, *en face* OCT shows the detailed pattern of choroidal vasculature in CSCR eyes [60, 75]. These dilated choroidal vessels are commonly localized within areas of increased choroidal vascular permeability when viewed on ICGA [73, 76]. By using *en face* OCT, abnormal hyper-reflective lesions at the level of Bruch's membrane and choriocapillaris layer were found to be concomitant with the presence of abnormal hypofluorescent areas at the late phase on ICGA [60, 77]. The presence of SRF and the abnormalities of RPE, which are common features of CSCR, can also be evaluated using OCT (Fig. 5).

In chronic cases, hyperreflective dots in SRF and the increase in number and duration indicate the prolongation of the disease duration [19, 60]. In addition, other OCT findings, such as photoreceptor elongation and defects in the inner or outer segment bands, may correlate with the prognosis of the final VA [78]. The disruption of the ellipsoidal band in cCSCR is evident, which highly correlates with VA loss and maybe a predictor of visual impairment [52]. OCT is also useful in detecting occult CNV by the presence of the double layer sign (DLS) which is characterized by an upper and lower reflective band that represent RPE and Bruch’s membrane, respectively [79]. Both DLS and pachydrusen can be found either in cCSCR or PCV, suggesting that CSCR may be a predisposing factor for PCV or proof of the same spectrum [40]. Significantly higher choroidal vascularity index (CVI; vascular area/total choroidal area on OCT scans) in eyes with aCSCR and its fellow eyes has been documented by several studies [80, 81].

### Optical coherence tomography angiography

OCTA is a non-invasive, depth-resolved imaging technique that assesses the changes in the retinal and choroidal vessels without any use of dye injection [82]. In CSCR, OCTA images often reveal areas with high signal intensity and abnormal dilation of choriocapillaris [19]. The presence of abnormal blood flow patterns, accompanied by surrounding hyperperfusion that indicated focal choroidal ischemia, has been detected on OCTA; this often parallels ICGA results in both acute and cCSCR [69, 83].

**Fig. 6:**
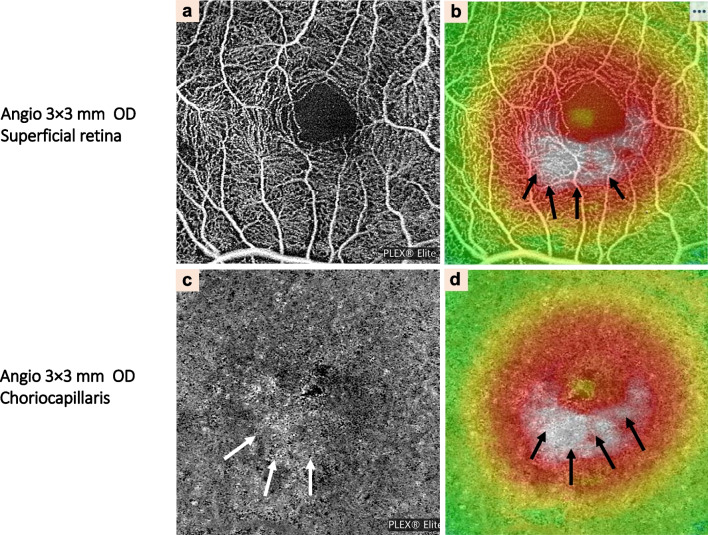
Clinical characteristics of central serous chorioretinopathy detected by optical coherence tomography angiography (OCTA). Automated segmentation of the superficial retina (at a range of 3 × 3 mm) showing foveal lesions on the topographic map. Focal protrusion of retinal pigment epithelium and high-density area of the choriocapillaris layer that corresponded to serous retinal detachment (black arrows) has also been noted (**a**, **b**). **c** Automated segmentation of the deep retina of the right eye. **d** Thickness map of automated segmentation of the deep retina of the right eye. The lesion which was showing by the white arrows in (**c**) and black arrows in (**d**) corresponded to serous retinal detachment which was indicated by black arrows in (**b**)

Other characteristic findings in the imaging of CSCR eyes are dark areas, dark spots, and abnormal choroidal vascularization. The dark areas that correspond to SRF appear as focal or diffuse, ill-defined, low-detectable flow areas while PED is characterized as black, single or multiple, well-delineated areas with no detectable flow at the level of choriocapillaris layer [60, 82]. Choroidal vasculature abnormality is seen as distinct, well-delineated, high-flow areas in the choriocapillaris layer, together with an abnormal dilation of choroidal vessels (Fig. 6). OCTA has several advantages in detecting macular neovascularization (MNV), especially occult MNV. Nonetheless, extra care and attention should be given when interpreting these typically large flat nets of type 1 NV observed in CSCR, which appear identical to MNV in some other pachychoroid diseases [82, 84].

Currently, several choroidal vasculature parameters detected by OCTA have been applied in clinical research as valuable biomarkers, including vessel density, fractal dimension, foveal avascular area, CVI, and area of flow signal voids (AFSV), and etc. The AFSV is significantly larger in unaffected eyes than in recurrent or persistent aCSCR [85]. Choriocapillaris Island (CCI) on OCTA was demonstrated in active CSCR underneath the area of neurosensory detachment, which represents the focal area of dysfunction of the affected choriocapillaris [86]. However, dark dots or areas in the choroidal capillary layer captured by OCTA can also be OCTA artifacts, and the presence of SRF is an important shadow-causing artifact in the choroidal capillary layer using SD-OCTA [87, 88]. Voids can still be detected in the choroidal capillary layer in CSC eyes with SRF by SS-OCTA, which can reduce the shadow artifacts caused by SRF [88].

### Fundus autofluorescence

Fundus autofluorescence (FAF), a non-invasive imaging modality, has been used to diagnose various RPE dysfunction diseases. FAF is related to lipofuscin changes within the RPE and outer retina and subretinal space alterations [89].

Some studies have shown that FAF imaging is useful in providing information for the evaluation of the retinal changes for CSCR compared to FFA. FAF uses two types of imaging techniques: short-wave FAF (SW-FAF) and near-infrared FAF (NIR-FAF) [60]. SW-FAF is thought to image the autofluorescence that originates from lipofuscin pigment, while NIR-FAF images from the melanin pigment of RPE and choroid. NIR-FAF has been proven to be a better predictor in identifying outer retinal changes in patients with CSCR than SW-FAF [90, 91].

FAF show a hyper-autofluorescence, a sign of RPE dysfunction and sometimes hypoautofluorescence in areas of atrophy. Meanwhile, in chronic cases, most of the eyes presented severe hypoautofluorescence corresponding to the leakage site noted in FFA (Fig. 2a); sporadically, hyper-autofluorescence can be observed in the neighbouring areas due to RPE dysfunction or atrophy [92]. Several studies have shown that different FAF represents a different clinical course and reflects the dysfunction of RPE and photoreceptors in CSCR: aCSCR exhibits decreased FAF intensity at the leakage points, hyper-autofluorescent, hypo-autofluorescent and minimal changes in the area of SRF; cCSCR shows discrete dots with hypo-autofluorescent; and mixed FAF patterns over areas of RPE atrophy can be detected in the sequelae of CSCR [89].

### Flavoprotein fluorescence

In recent years, a new, non-invasive technique–FPF has emerged as a sensitive biomarker for assessing retinal metabolic integrity. FPF analyses the metabolic tissue stress on mitochondria that occurs at disease onset, detecting early ocular dysfunctions before other signs or symptoms appear [93, 94]. Soon after the onset of retinal diseases, mitochondrial stress and early apoptotic responses in tissues induced by oxidants stress or other stimuli can be detected, suggesting that measurement of mitochondrial metabolic activity may serve as an early indicator of disease [95]. Therefore, a gradual increase of FPF levels can be seen when the level of oxidative stress increases, which is well correlated with a reduction in the mitochondrial membrane potential and increased risk of apoptosis [93, 94].

An abnormal elevation of FPF signals in eyes with diabetes, pseudotumor cerebri, and bilateral CSCR have been found [93, 95, 96]. These observations have led to the hypothesis that accumulations of flavoprotein may be a biomarker for early diagnosis of CSCR. A study that presented the FPF analysis involved three patients with unilateral CSCR to further investigate the utility of FPF as an indicator of CSCR-induced retinal metabolic stress. The patients were examined for FPF using an electron-multiplying charged-coupled device (EMCCD) camera. The average intensity of retinal FPF signals was measured. The results showed that the retinal FPF values of affected eyes with CSCR were significantly higher than unaffected eyes and normal control eyes [95]. Thus, FPF analysis may be a useful, rapid, and non-invasive technique in analysing the metabolic tissue stress of eyes with CSCR. However, further studies related to the use of FPF in the investigation of CSCR must be carried out due to limited pre-clinical or clinical evidence.

### Multispectral imaging system

**Fig. 7:**
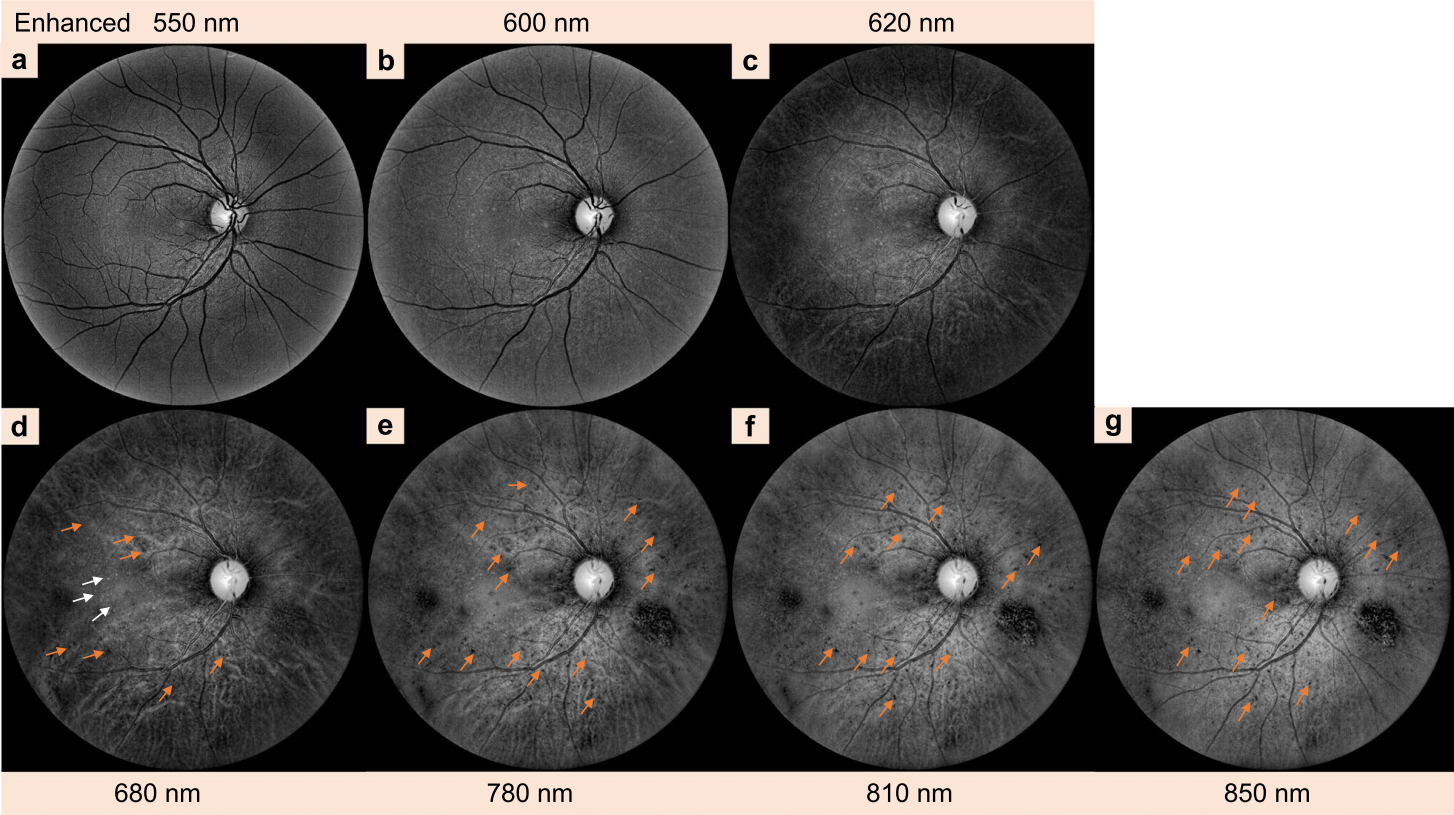
Clinical characteristics of central serous chorioretinopathy detected by multispectral imaging (MSI, C2000) in the right eye. MSI showing leakages of retinal pigment epithelium (RPE) in the right eye using different wavelengths (**a**–**g**). RPE leakages are characteristically observed as pinpoints of hypercyanescence at longer wavelengths of 680–850 nm (**d–g**); the focal hyperreflective areas (white arrows) and multifocal hyperfluorescent dots (orange arrows) shown in MSI corresponded well to the RPE leakage as detected by fundus fluorescence angiography

**Fig. 8:**
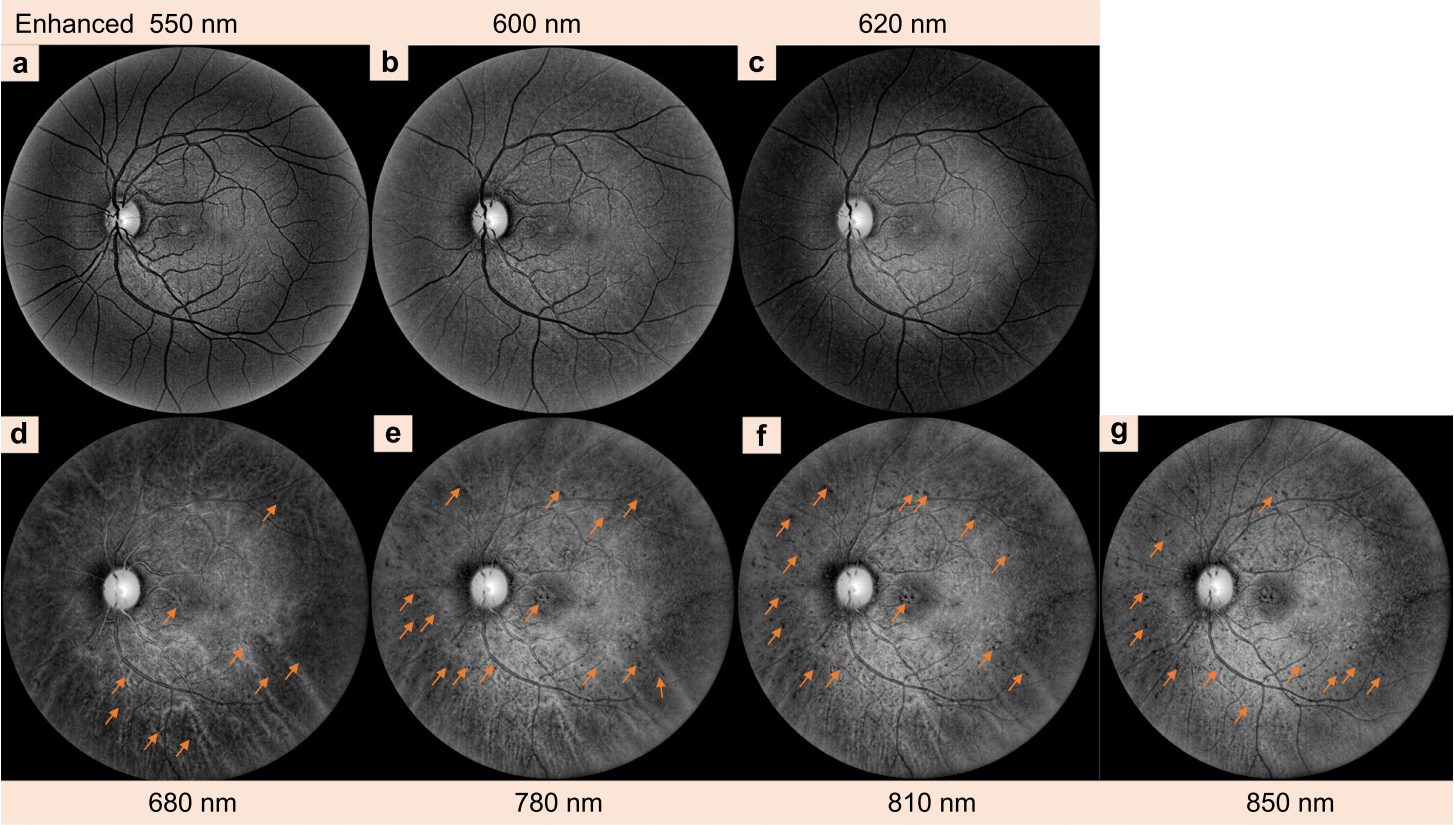
Clinical characteristics of central serous chorioretinopathy detected by multispectral imaging (MSI, C2000) in the left eye of the same patient using different wavelengths (**a**–**g**). MSI of the left eye shows the exact pinpoints (orange arrows, **d**–**g**) of hypercyanescence as the right eye at the wavelengths of 680 to 850 nm

In recent years, a non-invasive and light-emitting diode (LED)-based MSI system technique has also been applied to aid in the diagnosis and management of CSCR (Figs. 7, 8). MSI has been used in biomedical imaging such as in skin studies and pathological diagnosis [97]. MSI is also a novel and promising technique that allows for detailed visualization of the different layers of retinal and choroidal structures identified by different wavelengths (enhanced 550 nm, 600 nm, 620 nm, 680 nm, 780 nm, 810 nm, and 850 nm) [56, 98–100]. A study that involved 56 eyes with CSCR went through a series of clinical examinations including MSI, has suggested that RPE leakage and neurosensory detachment can be visualized by MSI instead of FFA. They concluded that MSI is a valuable adjunct to OCT and a possible non-invasive alternative to FFA [56]. However, the development of the MSI system in detecting CSCR needs to be explored in greater depth. Further studies are needed to determine the effectiveness of this method as a screening and diagnostic tool in patients with CSCR and whether it has the potential to replace FA and ICGA.

### Multicolour imaging

Multicolour imaging (MCI) is a confocal scanning laser ophthalmoscope-based imaging modality developed for Spectralis OCT that can demonstrate different retinal layers at wavelengths of 488 nm (blue, focuses on inner retinal and vitreoretinal interface, retinal fibre layer), 515 nm (green, focuses on deeper retina intraretinal exudative) and 820 nm (infrared, focuses on the outer retina and RPE). MCI is superior to the traditional colour, infrared and FAF images in interpreting parameters such as the boundary of the neurosensory retinal detachment and subtle changes in RPE and photoreceptors [101].

## Treatment

CSCR is considered one of the most common retinal diseases in clinical practice. With new updates in its management, several treatment modalities have been tailored for CSCR patients with different risk factors, clinical course, disease progression, and complications. aCSCR often resolves spontaneously, so observation is preferred [45, 102]. If observation failed, the possible treatment modalities can include PDT, traditional LP, SML, and oral medications as described in detail in the following sections. cCSCR is associated with a persistent disruption in VA; half-dose PDT is the most effective treatment for cCSCR in clinical practice, particularly in Asia. Furthermore, the management of psychopathology and psychosocial stress should be addressed during the recovery of CSCR. Discontinuation of corticoids is described in more details in the section on Risk Factors. Larger randomized control trials are required to validate the long-term efficacy and safety of the available CSCR treatment options.

### Observation

Observation is usually recommended as the first-line management for aCSCR. About 90%–95% of cases are typically self-limiting with spontaneous resorption of SRF [45]. However, interventions may be needed, such as in patients who experience persistent SRF, multiple occurrences, or patients who require rapid recovery of VA [103]. Observation without additional treatment within the first three months might be the preferred approach. Visual prognosis is good, and VA can be expected to recover to above 20% of the presenting VA once the issue has resolved. The gradual resolution of the neurosensory detachment can be observed by monitoring the patient from time to time. Studies have shown that vision recovery may be enhanced by risk factor modifications such as reduction or discontinuation of exogenous corticosteroid use where possible and reduction of other risk factors such as stress burden [46, 104], which we have described in detail in the pathogenesis and oral medications sections in this review. However, in chronic cases where SRF persists for more than 3–4 months or in patients with aCSCR who experienced numerous recurrences and sustained vision loss from a previous episode, treatment should be considered [45].

### Photodynamic therapy

When active intervention is indicated, PDT is introduced as an off-label treatment for both aCSCR and cCSCR and is a preferred option in Asia. PDT effectively reduces SRF, choroidal thickness and vascular hyperpermeability [45, 105]. PDT utilizes laser light (689 nm, 50 J/cm^2^) in combination with photosensitizer (verteporfin, Novartis, Switzerland) injection to treat both acute and cCSCR. PDT works by stopping abnormal leakages, allowing for the absorption of SRF, inducing vasoconstriction and choroidal vessel remodelling, and reducing choroidal hyperpermeability. During the process, free radicals is released when the verteporfin is activated to restore the damaged choroidal vessels as well as RPE.

Verteporfin PDT can be administered with full- or half-dose verteporfin. Positive results with reduced leakage, reduced choroidal thickness, and vasoconstriction of the abnormal choroidal vessels, were reported along with improved overall VA and without recurrence of CSCR [106, 107]. However, secondary CNV or post-treatment choriocapillaris hypoperfusion has been discovered months after PDT treatment. PDT also has the potential to induce treatment-related complications such as choriocapillaris closure and focal alteration of RPE, which can lead to photoreceptors damage [108, 109].

In order to minimize the occurrence of these complications, some safety-enhanced measures were adopted in recent years, such as the reduction of verteporfin dose or laser fluence [106]. Half-dose (3 mg/m^2^) or half-fluence (25 J/cm^2^) PDT was introduced to treat patients with cCSCR; half dose PDT is a better option with rapid reabsorption of SRF, resulting in a longer more effective and equal safety profile when compared with half-fluence PDT [45, 110]. A few studies uses one-third of the conventional PDT dosage to treat the patient with aCSCR [106, 111]. They have concluded that one-third-dose PDT is reliable and safe, leading to improved VA, reduced choroidal leakage, and accelerated resorption of SRF [105, 112, 113]. In a study comparing the effectiveness of half-dose PDT and one-third-dose PDT in chronic or recurrent CSCR, researchers found that one-third-dose PDT was more beneficial in terms of BCVA and CRT improvement at 12 months than half-dose PDT [114]. However, the optimal dosage of verteporfin PDT required to treat cCSCR has not been established yet. Rijssen et al. found that half-dose PDT is associated with fovea-involving atrophy [115]. Furthermore, the PLACE trial demonstrated that half-dose PDT is superior to high-density SML photocoagulation) in patients with cCSCR [116]. Table 1 summarizes the optional dosage of verteporfin and the efficacy of PDT from different studies.

**Table 1:** Clinical outcome from different doses and influence of PDT treatment

References	Study design	Participants	Dosage (Full dose or half, 1/3 dose)	Duration (Months, M)	Results
Chan et al. (2003) [108]	Prospective study	6 eyes with persistent or cCSCR	Full-dose PDT	12.7	Succeeding to stop fluorescein leakage without recurrence of CSCR
Chan et al. (2008) [106]	Prospective study	48 eyes with cCSCR	Half-dose PDT	12	Resolution of SRF and improvement of BCVA
Chan et al. (2008) [102]	Prospective study, RCT	63 eyes with aCSCR	Half-dose PDT vs. placebo	12	Absence of exudative macular detachment and improvement of VA
Zhao et al. (2009) [113]	Prospective study	15 eyes with aCSCR	70%, 60%, 50%, 40%, 30%, 20%, and 10% of full dose PDT	12	One-third-dose is safe and effective in the treatment of aCSCR
Bae et al. (2011) [117]	Prospective, a single pilot study	16 eyes with cCSCR	Low-fluence PDT vs. intravitreal injections of ranibizumab	3	Ranibizumab injections was not promising compared with low-fluence PDT in terms of anatomic outcomes
Uetani et al. (2012) [105]	Prospective study	16 eyes with cCSCR	Half-dose PDT vs. one-third-dose PDT	3	Half-dose PDT is more effective in resolution of SRF
Kim et al. (2014) [111]	Retrospective study	21 eyes with aCSCR	Half-dose PDT vs. observation	12	Rapid resolution of SRF in aCSCR by half-dose PDT
Kim et al. (2015) [118]	Retrospective study	57 eyes with cCSCR	Half-dose PDT vs. half-fluence PDT	24–62	CRT changes reflect PDT efficacy and predict long-term recurrence and early treatment outcomes
Fujita et al. (2015) [107]	Retrospective study	204 eyes with cCSCR	Half-dose PDT	12	Effective in treating SRD and BCVA improved significantly
Cheng CK et al. (2017) [110]	Prospective study, RCT	40 eyes with cCSCR	Half-dose PDT vs. half-fluence PDT	6	Both half-dose and half-fluence PDT were similarly effective in improving VA and SRF for cCSCR
van Dijk et al. (2018) [116]	RCT	179 patients with cCSCR	Half-dose PDT vs. high-density subthreshold micropulse laser (HSML)	8	Half-dose PDT is superior to HSML for treating cCSCR
Hua et al. (2018) [[112]	Retrospective study	68 eyes with cCSCR	One-third dose PDT	6	Improvement of BCVA, reduces choroidal vasodilatation and leakage, accelerates absorption of SRF, and prompt recovery of photoreceptors
van Rijssen et al. (2021) [115]	Retrospective study	34 eyes with cCSCR	Half-dose PDT	12	SRF and BCVA were improved
Pichai et al. (2021) [[114]	Retrospective study	46 eyes with recurrent or cCSCR	Half-dose PDT vs. one-third-dose PDT	12	One-third-dose PDT was effective in improving BCVA and CRT but had higher recurrence rate of SRF

*aCSCR* = acute chronic central serous chorioretinopathy; *cCSCR = *chronic central serous chorioretinopathy; *BCVA* = best corrected visual acuity; *CRT = *central retinal thickness; *PDT = *photodynamic therapy; *RCT* = randomized controlled trial; *SRD* = subretinal detachment; *SRF* = subretinal fluid; *VA* = visual acuity

To establish better efficacy outcomes and longer-term safety, more extensive studies of PDT with long-term follow-up are still needed. The ideal dosage and fluence of PDT treatment would be the one that shows the best efficacy and safety for the CSCR patient with the least possible complications.

### Laser photocoagulation

Conventional LP was the only treatment available for CSCR before the introduction of PDT. Low intensity (level 1 retinal tissue reaction), short exposure duration (0.1 s), and 50 µm laser burns are the common exposure parameters. The thermal laser is applied directly to the focal sites of RPE leakage evident on FFA. Argon laser is widely used at the moment while the other types of laser used previously were xenon and krypton [33]. The exact mechanism of action of LP is not fully understood but it is believed to “seal” the leakage site, to prevent further leaking, and to photocoagulate the cluster of diseased RPE cells. It also activates the pumping function of surrounding RPE cells to direct fluid back into the choriocapillaris.

LP is recognized to be effective for both acute and cCSCR [119, 120]. Application of LP to the leakage site will help to resolve SRF and restore the VA effectively in a short time, which has been demonstrated in several studies [45, 121]. Some of the aCSCR cases cannot be treated by LP as the leakage site is often very close to the fovea, or the size of the leakage point is too big and difficult to recognize [102, 122]. LP can only be applied if the leakage point is located more than 400 μm from the foveal center. However, even if LP is applied appropriately, it can cause side effects such as iatrogenic anatomical and functional chorioretinal damage caused by the whitening burn of the retina. Laser-induced CNV and scotoma can also occur. Resolution of the serous neuroepithelial detachment and anatomical recovery of macular within 3–4 months of disease onset is an optimal therapeutic target as prolonged detachment is associated with photoreceptor atrophy [120]. Thus, conventional LP should not be used as the first-line treatment for CSCR. More studies and clinical trials need to be carried out to prove their significant benefits for CSCR patients. Long-term follow-up is also necessary to determine any complications such as CNV.

### Subthreshold micropulse laser photocoagulation

Besides argon laser therapy, SML is another type of laser treatment that is used to treat CSCR eyes with identifiable leakage points. This high-density laser uses subthreshold energy in a burst or “envelop” of micropulse which selectively targets the RPE cells without leaving scar tissue to minimize the retinal damage [123, 124]. Multiple and overlapping invisible spots that can only be microscopically and histologically detected are produced by this treatment, which is adequate to restrict the photothermal effect to the RPE without affecting the neurosensory retina. The 5%–10% duty cycle of SML is set by the ophthalmic surgeons. There are several wavelengths of pulse lasers used in SML to target different cells in the retina such as green 532 nm laser, yellow 577 nm laser, and infrared 810 nm laser [125].

A clinical trial that utilized yellow 577 nm SML in treating non-resolving CSCR has concluded that the resolution of SRD can be accelerated in cCSCR, with significant functional improvement and without leaving any laser-induced lesions. A retrospective study also found that among all the patients with multiple spots treated with yellow 577 nm SML, 79% had a reduction in the macular thickness, and no complications such as RPE atrophy, CNV, choroidal hypoperfusion and transient reduction of macular function were found [126]. SML is relatively safe to apply close to the fovea as it is poorly absorbed by the macular xanthophyll pigment [124, 127].

On the other hand, SML treatment targets the RPE without improving choroidal thickness which is a marker of choroidal hyperpermeability. Therefore, half-dose PDT, which has been found to be effective in reducing the choroidal thickness is thought to be a more optimal option in preventing the recurrence of CSCR [118]. It was also shown in the PLACE trial that half-dose PDT is preferable for patients with cCSCR compared to SML, as the percentage of patients with completely resolved SRF and improvement in BCVA were significantly higher [116]. More clinical trials are warranted to investigate the significance of its efficacy and safety including comparison with PDT and conventional LP.

### Oral medications

Oral medications may be an option for patients who are not suitable (such as extensive SRD or unable to identify leakage points) or reject invasive treatment modalities such as PDT, LP, and intravitreal anti-VEGF. Several medications have been used in patients with CSCR, such as spironolactone, eplerenone, rifampicin, 5-alpha reductase inhibitors, aspirin, acetazolamide, beta-blockers, and vitamins [128, 129]. The clinical outcome of randomized control trials (RCTs) involving these drugs is controversial.

Both spironolactone and eplerenone are mineralocorticoid antagonists that inhibit aldosterone and glucocorticoids from binding to mineralocorticoid receptors [128]. One of the risk factors associated with CSCR patients is the high levels of endogenous and exogenous corticosteroids. It is believed that the predominant pathway in CSCR is the mineralocorticoid pathway [20, 128]. A study in mice showed that choroidal and retinal vasculature express glucocorticoid and mineralocorticoid receptors [21]. Several retrospective and prospective studies on the inhibition of mineralocorticoid receptors have been carried out by using oral spironolactone and eplerenone as potential treatment methods for CSCR patients. Most results have shown positive outcomes demonstrating a significant reduction of SRF, decreased central macular thickness (CMT), and improvement in VA [57, 130, 131]. Although oral spironolactone is not a first-line treatment, it is helpful for patients with non-resolving CSCR with SRD [132]. The VICI study found that oral eplerenone (25 mg/day for 1 week then increasing to 50 mg/day for up to 12 months) was not superior to placebo for improving VA in people with cCSCR at 12 months [133]. The multicenter, open-label, randomized controlled trial SPECTRA study demonstrated that half-dose PDT is superior to oral eplerenone for cCSC in terms of both short-term safety and efficacy outcomes [134]. However, as the treatment dosage, duration, primary outcome measure and sample size vary from study to study, more studies are required for better evaluation. This treatment option could offer a new approach for CSCR patients, especially in chronic cases. Furthermore, both VICI and SPECTRA have been designed to investigate the effectiveness of different treatment modalities for cCSCR; it would be interesting to determine whether early administration of eplerenone could improve the clinical outcome of aCSCR. To date, there are no studies looking into the use of eplerenone in aCSCR.

Rifampicin, an antibiotic used to treat tuberculosis and other bacterial infection, is thought to favourably alter the metabolism of endogenous glucocorticoids, and thus lead to an improvement in CSCR manifestations [135]. A case study of rifampin in a tuberculosis patient who also had cCSCR demonstrated SRF resolution after the commencement of rifampicin. Rifampicin induces the production of cytochrome P-450 in the liver with enhanced hepatic metabolism of endogenous corticosteroids [136]. However, close follow-up is important during therapy as there is a risk of rifampicin-induced hepatotoxicity [137].

Finasteride is a type of 5-alpha reductase inhibitor that inhibits type II 5-alpha reductase to prevent the synthesis of dihydrotestosterone (a potent androgen) from testosterone, in which dihydrotestosterone has a high affinity to the androgen receptors. As androgens are believed to play a role in the pathogenesis of CSCR, antagonists such as finasteride may theoretically be an aid to treatment [128, 138, 139]. In a pilot study, five patients with cCSCR were administered 5 mg of finasteride daily for three months. The result revealed that finasteride had a remarkable effect in the reduction of mean SRF and CMT after six months, suggesting its potential therapeutic effect in the initial treatment of patients with chronic CSCR. However, it was also found that some patients experienced a recurrence of SRF after the discontinuation of finasteride. Therefore, long-term and larger clinical trials are needed to further assess its efficacy and safety in CSCR.

Some studies showed an increased level of plasminogen activator inhibitor 1 (PAI-1) in CSCR patients compared to the control group, suggesting that hypercoagulability might play a role in the pathogenesis of CSCR [140, 141]. Aspirin has anti-aggregation effects and is effective in reducing the serum levels of PAI-1 [128, 141]. In a pilot study, patients with CSCR were given 100 mg of aspirin daily for 1 month then on alternating days for 5 months; low-dose aspirin rapidly improved VA with a lower recurrence rate in CSCR patients compared with the untreated control group [142]. However, due to limited data, further trials are required.

Acetazolamide, a carbonic anhydrase inhibitor used as a treatment for idiopathic intracranial hypertension and glaucoma, has also been utilized in CSCR. It exerts this effect in the RPE, thus facilitating resorption of SRF and restoring normal polarization in the RPE. It also can restrain ocular glutamyl transpeptidase activity by preventing the depletion of glutathione that is caused by glutamyl transpeptidase [128, 143–145]. It has been proven by a few studies for its efficacy in the resolution of macular oedema caused by various intraocular pathologies or surgery [146, 147]. In a prospective, nonrandomized, comparative trial using oral acetazolamide, the time for both subjective visual function improvement and SRF resolution were shorter than the control group without affecting the final BCVA or recurrence rate [148]. However, in a recent study by Kwak et al., it was suggested that acetazolamide does not have any impact on eyes with CSCR after three months except for hastening SRF resorption [149]. Further investigations are needed to evaluate the use of acetazolamide in CSCR.

The hypercoagulable state is proposed to be one of the risk factors for CSCR. Oral intake of Aspirin (100 mg daily for the first month then 100 mg every other day for 5 months) was shown to improve VA rapidly and reduce the occurrence effectively by increasing plasminogen activator inhibitor 1 (PAI-1) in an RCT [150]. A large cohort is needed to further confirm the efficacy.

Melatonin has been involved in the regulation of sleep, seasonal disorders, and aging. Melatonin was also found to have antitumoral properties and neuroprotective effects. In a prospective comparative series, 13 patients with cCSCR were given 3 mg melatonin tid, and five patients with placebo. At the one month follow-up, 87.5% of patients with oral melatonin have their VA improved, indicating melatonin is safe, well tolerated, and effective in treating cCSCR. Another study also reported that melatonin and eplerenone were equal in improving BCVA in cCSCR patients, melatonin was superior in promoting the reabsorption of SRF [151–154]. However, a large-scale prospective RCT is warranted.

### Anti-vascular endothelial growth factor agent

Anti-vascular endothelial growth factor (anti-VEGF) injection has also been used to treat both aCSCR and cCSCR but with conflicting data. Anti-VEGF therapy has been shown to effectively reduce choroidal hyperpermeability in patients with CSCR, although the expression level of VEGF is not elevated in the aqueous humour [45, 155]. Some studies have shown that anti-VEGF therapy leads to similar clinical outcomes in aCSCR in terms of BCVA when compared with observation alone. However, the foveal ellipsoid zone was more frequently preserved in CSCR eyes treated with anti-VEGF [156]. A single-center retrospective comparative study has shown that the resolution time of SRF can be shortened by using ranibizumab and bevacizumab on aCSCR, although neither intravitreal bevacizumab nor ranibizumab had a positive effect on the improvement of the BCVA [157]. A recent meta-analysis of 14 studies to determine the clinical efficacy of anti-VEGF found that anti-VEGF had no advantages in aCSCR at 6 months in terms of BCVA and CMT. However, significant differences were observed in CMT reduction for chronic cases but there was no difference in the final BCVA between the treatment and observation groups. Taken together, anti-VEGF therapy may be a viable alternative for cCSCR treatment [129].

Although anti-VEGF is being used as a treatment for CSCR patients, studies have been carried out to compare the efficacy and safety between PDT and anti-VEGF therapy in CSCR. An RCT has shown that half-fluence PDT may be superior to three monthly doses of intravitreal ranibizumab. VA was improved in both groups, but the PDT group showed significantly better results than the anti-VEGF group [117, 158]. Anti-VEGF agents have a more clearly defined role in treating CSCR-related CNV.

### Topical NSAIDs and carbonic anhydrase inhibitors

In recent years, several pieces of clinical evidence have shown that prostaglandins and aldosterone are involved in the pathogenesis of CSCR. Topical non-steroidal anti-inflammatory drugs (NSAIDs) have been applied routinely in the peri-operate period to prevent or treat surgical-related macular oedema by inhibiting cyclooxygenase (COX-1 and COX-2) enzymes, to decrease the production of prostaglandins and their downstream inflammatory factors. Rapid improvement of BCVA in topical NSAIDs or treated CSCR eyes was found in either case reports or cohort retrospective studies [159].

Anhydrase inhibitors have also been shown to be effective in improving the absorption of the SRF. Dorzolamide is a human carbonic anhydrase II inhibitor. Topical dorzolamide results in a more rapid reduction of CMT in comparison with observation in a prospective RCT. Currently, most studies suffer from small sample sizes and are short term in nature hence, a large and long-term cohort study is warranted to confirm that carbonic anhydrase inhibitors may be a novel treatment for cCSCR.

## Conclusion

The emergence of new imaging modalities such as OCT and OCTA have revolutionized our understanding of the pathogenesis and development of CSCR. Currently, the interpretation of CSCR mostly depends on the advances of evidence-based imaging, the continuous progress in pathology and molecular biology will lead to a deeper understanding of the pathogenesis of CSCR. CSCR is continuously updated based on the understanding of the pathogenesis of pachychoroidal spectrum disorder and venous overload choroidopathy. Therefore, a deep understanding of the pathogenesis of pachychoroidal spectrum disorder and venous overload choroidopathy is important to support the clinical discovery of new molecular biological markers and therapeutic targets in ophthalmology.

## Data Availability

All data generated or analysed during this study are included in this published article.

## Abbreviations

AMD: Age-related macular degeneration
aCSCR: Acute central serous chorioretinopathy
BCVA: Best corrected visual acuity
CCI: Choriocapillaris Island
CMT: Central macular thickness
CNV: Choroidal neovascularization
CSCR: Central serous chorioretinopathy
cCSCR: Chronic central serous chorioretinopathy
CCSF: Carotid cavernous sinus fistulas
CVI: Choroidal vascularity index
DLS: Double layer sign
FAF: Fundus autofluorescence
FFA: Fundus fluorescence angiography
FPF: Flavoprotein fluorescence
*H. pylori*: *Helicobacter pylori*
ICGA: Indocyanine green angiography
LP: Laser photocoagulation
MCI: Multicolour imaging
MNV: Macular neovascularization
NSAIDs: Non-steroidal anti-inflammatory drugs
OCT: Optical coherence tomography
OCTA: Optical coherence tomography angiography
PCV: Polypoidal choroidal vasculopathy
PDT: Photodynamic therapy
PED: Pigmented epithelial detachment
PSQI: Pittsburgh sleep quality index
RPE: Retinal pigment epithelium
RVO: Retinal vein occlusion
SML: Subthreshold micropulse laser
SRF: Subretinal fluid
VA: Visual acuity
